# Age-Related Changes of the Ocular Surface: A Hospital Setting-Based Retrospective Study

**DOI:** 10.1155/2014/532378

**Published:** 2014-08-11

**Authors:** Laura Ottobelli, Paolo Fogagnolo, Marta Guerini, Luca Rossetti

**Affiliations:** Eye Clinic, Dipartimento di Scienze della Salute, San Paolo Hospital, University of Milan, 20142 Milan, Italy

## Abstract

*Purpose*. To investigate the effects of age on the prevalence of ocular surface diseases (OSD), adherence to treatment, and recovery rates. *Patients and Methods*. Retrospective analysis of 3000 clinical records from a first-level general ophthalmology clinic. Patients with OSD were prospectively submitted a questionnaire to assess compliance and recovery rates. *Results*. OSD prevalence was 10.3%. Patients with OSD were significantly older than patients without it: 67.5 ± 20.3 versus 57.0 ± 22.0 years (*P* = 0.036). No significant difference in season distribution was shown. Dry eye disease (DED) represented 58% of OSD; its prevalence increased with age until 80 years old and suddenly decreased thereafter. Asymptomatic DED was 37%. Adherence to treatment in OSD was very high (94%); recovery rates were lower in patients aged 21–40 and 61–80 (resp., 65.5% and 77.8%) and this was associated with higher OSDI scores. Tear substitutes represented 50% of all prescribed medications; their use increased with age. *Discussion*. In a “real-life” low-tech setting, OSD showed a prevalence of 10.3%. DED was the most prevalent disease, and it was asymptomatic in more than 1/3 of cases.

## 1. Introduction

The ocular surface system (OSS) is defined as the wet-surfaced epithelia of the cornea, conjunctiva, lacrimal gland, accessory lacrimal glands, nasolacrimal duct, meibomian gland, and their apical and basal matrices, linked as a functional system by both continuity of epithelia, by innervation, and the endocrine and immune systems. Several age-related changes occur in most components of the OSS: meibomian glands decrease in density and their ducts may keratinize causing occlusion and consequent alteration in lipid secretion; lacrimal gland secretion diminishes and its composition changes; corneal nerve density decreases [1–5]. These changes are frequently associated with inadequate volume of tears, tear film instability, increased evaporation, and abnormal immune responses. Tear film impairment is therefore associated with a number of different ocular surface diseases (OSD), which may range from dry eye disease (DED) to infections and immune diseases [6].

All these conditions negatively impact quality of life (QoL). Assessment of the vision-related QoL is important in the management of patients suffering from OSD: it reflects the burden of disease experienced by the patient, overall assessing the impact of OSD on the individual and it helps monitor the changes occurring in patients with OSD. The Ocular Surface Disease Index (OSDI) is a 12-item questionnaire designed to provide a rapid assessment of the symptoms of OSD consistent with chronic dry eye, its severity, and its impact on vision-related functioning.

OSDI has been shown to have a high reliability, reproducibility, and validity [7]. It correlates weakly but positively with objective markers of OSD such as tear film break-up time, Schirmer test, and lissamine green surface staining. It has been validated with good sensitivity and specificity to detect normal subjects and patients with a value of ≤12; scores 13 or greater indicate OSD [8, 9].

Epidemiology studies of OSD are available only for DED and allergic conjunctivitis. DED has an extremely variable prevalence, as it is reported to affect 5 to 34% of the population, lying close to the higher bound of the range in subjects 50 years old or more. Females are more affected than males (17.0% versus 11.1% [10]), having nearly a 3-fold higher risk of DED [11–13]. Allergic conjunctivitis has been reported to range from 10 to 20% of the population [14].

To the best of our knowledge there are no studies exploring the effect of age on the prevalence of OSD. Therefore, through a hospital setting-based review of first-level general ophthalmology visits, we investigated the association of OSD and age as well as either patient's adherence to treatment and recovery rates or therapy and eye-drop instillation procedure efficacy based on patient's age.

## 2. Materials and Methods

This was a retrospective cross-sectional study with a prospective part consisting of the administration of a questionnaire on the group of patients with OSD. It was conducted at the Eye Clinic of San Paolo Hospital of Milan between January and December 2012.

**Figure 1:**
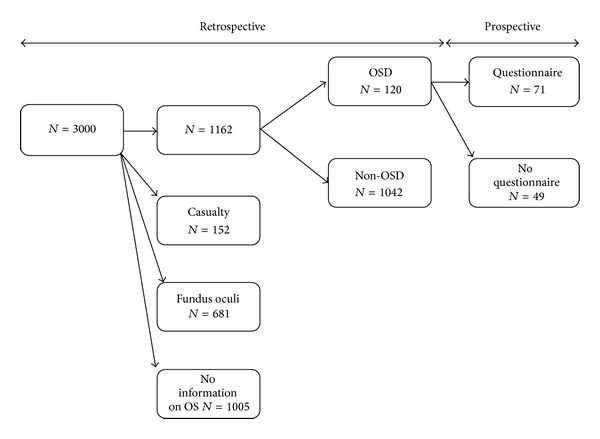
Flow chart of the events of the study.

3000 clinical records obtained from a first-level general ophthalmology clinic (the first 250 per month) were analysed and divided into 4 groups based on the type of visit (Figure 1): 1010 first ophthalmic visits (33.7%), 1157 control visits (38.6%), 152 access from emergency department (5%), and 681 fundus oculi examinations (22.7%).

Included were patients undergoing first ophthalmic visits and control visits. Exclusion criteria were emergency department visits, fundus oculi examinations, and inability to retrieve information of the ocular surface from the medical chart. The presence of ocular or systemic comorbidities was not an exclusion criterion as well as the medical prescriptions received at the end of the visit.

After applying exclusion criteria, the original dataset was restricted to 1162 patients, whose main characteristics are summarized in Table 1.

OSD was diagnosed in the presence of one or more of the following:

1. one or more of the following symptoms related to OSD: dryness, grittiness, burning, foreign body sensation, and visual disturbance;
2. tear film abnormalities (Schirmer *I* test results ≤ 5 mm/5 minutes or tear film break-up time < 10 seconds);
3. any ocular surface abnormality.

The prevalence of OSD was calculated, and sex, age, seasonality, diagnosis, and therapeutic prescriptions were recorded.

Within one month after the visit, patients with OSD were contacted by phone and submitted a questionnaire to assess their adherence to the therapy, the persistence/disappearance of symptoms, and their OSDI score. OSDI was normal (0–12 points) or showed mild (13–22 points), moderate (23–32 points), or severe (33–100 points) disease.

With the questionnaire, we inspected the adherence to treatments; the ability to self-administer treatments and to instill the proper dosage into the eyes; treatment-related satisfaction; and the management of persistent symptoms. 71 out of 120 subjects suffering from OSD answered the questionnaire, 9 refused, 4 did not understand Italian and English language, 1 had died, 29 patients could not be contacted, and 6 patients were not called because they were recommended a surgical treatment.

## 3. Results

The prevalence of OSD was 10.3% (120/1162).

The mean age for subjects without OSD was 57.0 ± 22.0 years (range: 1–100) and for OSD 67.5 ± 20.3 years (range: 3–84; *P* = 0.01), the ratio between females and males was 64/56.

**Figure 2:**
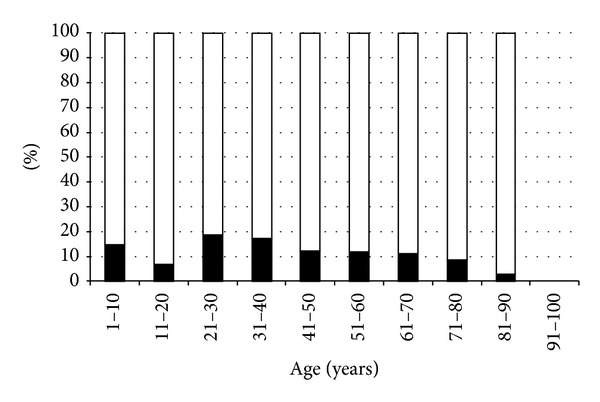
Percentage of subjects suffering and not suffering from OSD by age group. Black bars: presence of OSD; white bars: absence of OSD.

OSD prevalence was equally distributed from childhood to the middle old age, decreasing after 80 years old (Table 2, Figure 2), without significant differences in seasonal distribution.

We classified OSD patients in 5 categories: DED (58.3%), infections (19.2%), allergies (15.8%), eyelid pathologies (3.3%), and trauma (3.3%). The distribution of the etiologies was different at varying ages: infections showed a constant distribution across all age groups; about 75% (14/19) of the cases of allergy affected subjects younger than 60; and eyelid pathologies equally affected both the young and the elderly, but the causes were different (hordeolum and chalazion were more prevalent under 40 years of age, whereas blepharitis was progressively increasing from 30 years of age). Few traumas were reported, and they also occurred in two age groups based on different etiologies: foreign bodies in the class of 21–40 years; falls in the classes of 71–100 years.

**Figure 3:**
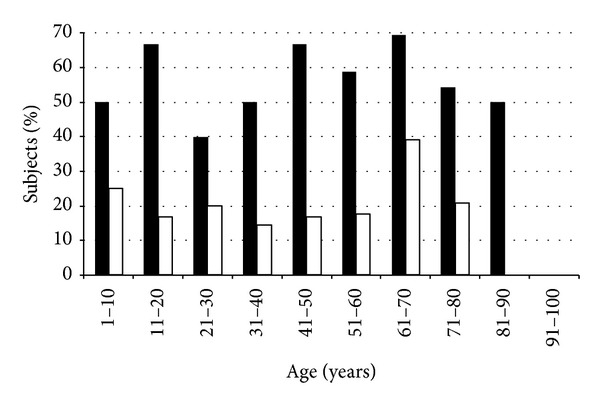
Percentage of overall and asymptomatic dry eye in OSD patients by age group. Black bars: overall dry eye; white bars: asymptomatic dry eye (OSDI ≤ 12).

DED represented 58% (70/120) of OSD in the population. The number of cases with DED increased with age until 80 years old, and it suddenly decreased for patients with 81 years old or more (Figure 3). DED was distinguished in two groups: asymptomatic (OSDI ≤ 12) and symptomatic (OSDI > 13); in our dataset, 37% of subjects had asymptomatic DED, with a percentage apparently increasing over 60 years of age (Figure 3). Female/male ratio for DED was 38/32.

**Figure 4:**
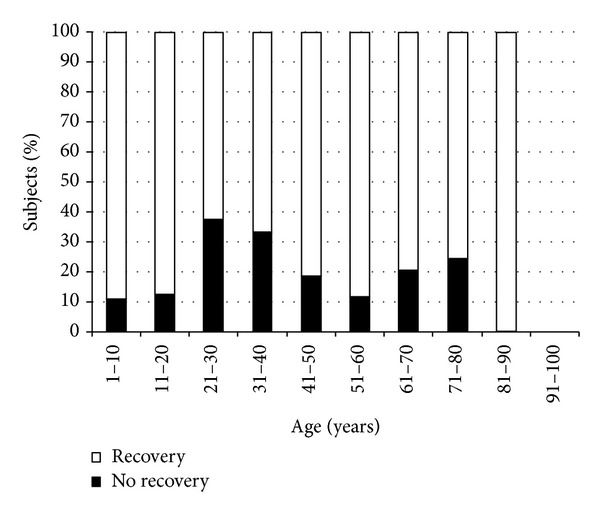
Percentage of OSD recovery rates by age group.

Recovery rates for OSD ranged from 60 to 100% depending on age (Figure 4).

Compliance was high among the 71 patients submitted to the questionnaire, as 67 (94%) followed the medical therapy as prescribed by the ophthalmologist. Lack of resolution occurred in 11/67 patients (16.4%); these patients asked a second opinion (39%), required further control visits (21%), underwent surgical treatment (11%), modified the therapy without consulting the doctor (7%), tolerated symptoms (4%), or recovered without any medical therapy (18%). Only 4 patients (6%) did not adhere to the prescribed treatment either because they spontaneously recovered (50%) either because of the onset of an allergic reaction (25%) or a lack of faith in the doctor (25%). Unexpectedly, most of the nonadherent patients were graduated (95.9%); no correlation between age and adherence to treatment was found.

**Figure 5:**
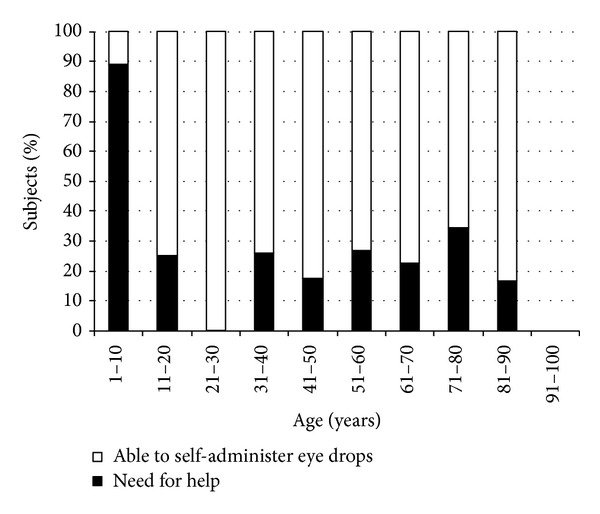
Percentage of subjects able and not able to self-administer eye drops by age group.

Patients were also asked if they were able to self-administer eye drops: 28.4% needed to be helped—especially subjects younger than 10 and aged 71–80 years old (88.9% and 34.3%, resp.)—and most of them reported a difficulty in aiming a drop onto their eye (Figure 5). Nevertheless, 92% patients never forgot to put eye drops in.

**Figure 6:**
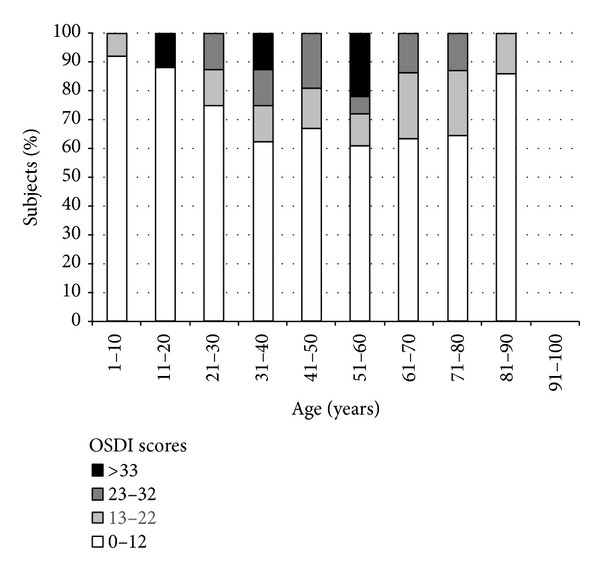
Percentage of OSDI scores by age group.

OSDI scores were usually ≤12, but the higher percentages of OSDI scores >13 were found in subjects aged 31–80 years old (Figure 6).

**Figure 7:**
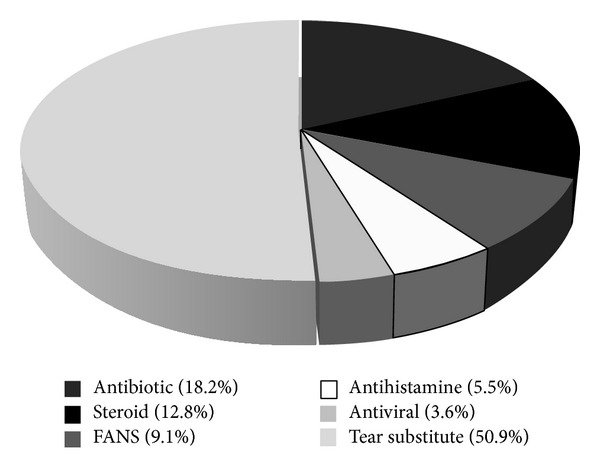
Therapy distribution.

**Figure 8:**
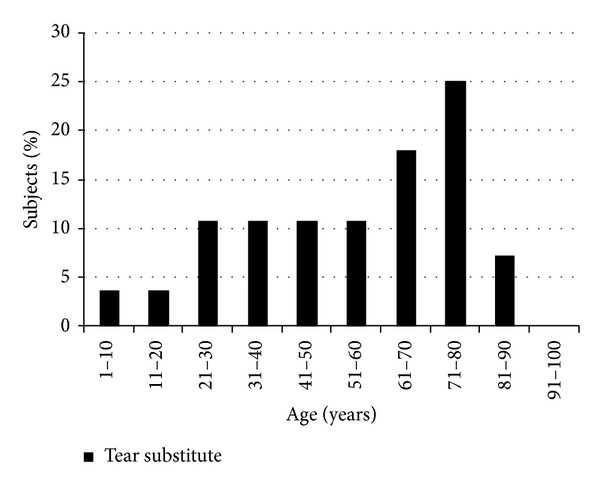
Percentage of tear substitutes by age group.

Therapy prescription in OSD was considered: tear substitutes represented 50.9% of the topical medications, followed by antibiotics (18.2%), steroids (12.8%), FANS (9.1%), antihistamines (5.5%), and antivirals (3.6%) (Figure 7). Tear substitutes were increasingly prescribed with increasing age, with a peak for patients aged 61–80 years old (Figure 8).

## 4. Discussion

The results of studies on epidemiology of a disease depend mostly on two factors: the features of the population and the diagnostic criteria and instruments used for diagnosis.

This paper included a relatively small sample of 1162 patients, representative of the population of subjects seen on a first-level general ophthalmology clinic. We collected a similar number of consecutive cases per month over a 1-year interval, so that the dataset is not influenced by seasonal variations of diseases. The study was a retrospective evaluation of routine visits performed using slit-lamp examination and, at discretion of the physician, dying with fluorescein to evaluate parameters related to dry eye (break-up time, epithelial staining, and clearance of fluorescein). Due to the use of low-tech methods, underestimation of conditions such as DED, particularly in asymptomatic cases, is extremely likely; moreover, the population of this study is not fully representative of the whole population, as in our clinic, patients with glaucoma or corneal pathologies (who have a very high prevalence of OSD [12, 15, 16]) are directly referred to tertiary services. Nevertheless, we found a prevalence of OSD of about 10%, which is confirmatory of the literature. Patients with OSD were significantly older than patients without it [6, 12, 13].

Unexpectedly, the distribution of OSD per decade was different than reported in the literature, as we found that the prevalence of OSD was higher in patients with 20 to 40 years. This data is influenced by the different prevalence of allergy over age [14, 17], the effect of working activity (the study included patients with foreign body), and the relatively higher-than-expected prevalence of DED in young patients [13, 18]. This prevalence is possibly due to the large prevalence of video-terminalists and users of contact lenses [19–22], a fact that is also linked to the lower rate of recovery rate shown in Figure 4.

The prevalence of OSD suddenly decreased after 80 years of age, a fact that may be explained considering that patients tend to consider this problem as “minor” when compared to general diseases and may not require ophthalmic evaluation. Eye doctors might also tend to overlook mild cases on this group of patients (as a matter of fact, the prevalence of asymptomatic DED, which peaked in the interval of 60–80 years of age, was reported as null over 80 years).

The major cause of OSD was, as expected, DED, which affected about 60% of patients with OSD, and a mild but constantly higher prevalence was found for females of age between 20 and 80 years. OSD influence on QoL was indirectly estimated through OSDI score: the higher the score, the worse the QoL [23, 24]. OSDI had a peak of severity in the decade 51–60, even if data in the range of ages of 31–80 were overall similar.

The adherence to treatment and the recovery rates found in this paper were higher than previously reported [25, 26]. The lack of adherence in graduated patients with OSD is possibly linked to the awareness that OSDs are frequently benign and self-limiting.

Eye drops prescription increased with age, with a peak for patients aged 61–80 years old. Tear substitutes were the most frequently administered treatment (more than 50% of all treatments): formulations containing sodium hyaluronate and/or carboxymethylcellulose were the most commonly prescribed, thus reflecting efficacy data from several studies [27–29]. Preservative-free eye drops were introduced to reduce toxic or allergic reactions when applied to the injured ocular surface; treatment with topical coenzyme Q10 also improved ocular surface stability [30].

In conclusion, this paper reported the prevalence of OSD in a group of patients visited with low-tech instruments in a “real-life” setting. The prevalence found was about 10%, with DED being the most prevalent disease (60% of cases). We investigated the distribution of different OSDs at varying ages, the prevalence of asymptomatic disease, and the implications on the efficacy and distribution of treatments as well as the adherence to them.

## Figures and Tables

**Table 1:** Demographic and ophthalmic characteristics of study participants.

	Overall	OSD	NO OSD
Number of patients	1162	120	1042
Age, years (SD)	61.3 (21.2)	67.5 (20.3)	57.0 (22.0)
Sex, F/M	616/546	64/56	552/490
Refraction, diopters (SD)	−1.20 (1.9)	−1.13 (1.7)	−1.31 (2.1)
IOP, mmHg (SD)	15.6 (4.2)	15.5 (3.9)	15.7 (4.4)

**Table 2:** Prevalence and distribution of OSD etiology by age group.

Age (y)	Overall	OSD	F/M	Infection	Allergy	Eyelid pathology	Trauma	Dry eye	OSDI < 12
1–10	27	4	2/2	1	0	1	0	2	1
11–20	87	6	3/3	0	2	0	0	4	1
21–30	53	10	6/4	3	1	1	1	4	2
31–40	82	14	6/8	0	5	0	2	7	2
41–50	148	18	10/8	3	2	1	0	12	3
51–60	143	17	9/8	3	4	0	0	10	3
61–70	205	23	13/10	5	2	0	0	16	9
71–80	277	24	13/11	6	3	1	1	13	5
81–90	132	4	2/2	2	0	0	0	2	0
91–100	8	0	0	0	0	0	0	0	0

	1162	120	64/56	23	19	4	4	70	26
